# Neutralizing antibodies induced by IpaB and IpaC mRNA vaccines inhibit *Shigella flexneri* invasion

**DOI:** 10.1128/spectrum.00993-25

**Published:** 2025-09-02

**Authors:** Ye-Eun Moon, Timothy An, Pil-Gu Park, Kee-Jong Hong

**Affiliations:** 1 Department of Health Sciences and Technology, GAIHST, Gachon University 216845, Incheon, Korea; 2 Department of Bio-medical Sciences, GAIHST, Gachon University 216845, Incheon, Korea; 3 Department of Life Science, Gachon University 65440, Seongnam, Korea; 4 Department of Microbiology, Gachon University 216845, Incheon, Korea; 5 Lee Gil Ya Cancer and Diabetes Institute, Gachon University 216844, Incheon, Korea; 6 Korea mRNA Vaccine Initiative, Gachon University 65440, Seongnam, Korea; Icahn School of Medicine at Mount Sinai, New York, New York, USA

**Keywords:** shigellosis, multiple serotypes pathogen, conserved protein, mRNA vaccine

## Abstract

Shigellosis, caused by *Shigella* species, remains a significant global health burden due to its high morbidity and mortality, particularly in developing countries, and frequent antimicrobial resistance. Thus far, various *Shigella* vaccine candidates have been tested, but many have shown limited immunogenicity, high reactogenicity, or insufficient cross-serotype protection due to serotype-specific O-polysaccharide (OPS) targeting. In this study, we evaluated mRNA vaccine candidates targeting the highly conserved *Shigella* invasion plasmid antigens (IpaB and IpaC), aiming to provide a preventive strategy that can address various serogroups of *Shigella*. Using a mouse intraperitoneal infection model, we assessed the immune responses and protective efficacy of lipid nanoparticle (LNP)-encapsulated mRNA vaccines. The results demonstrated that the vaccine induced neutralizing antibodies capable of inhibiting intracellular invasion by *Shigella flexneri*, confirming its potential for protection against this serotype.

**IMPORTANCE:**

This study aimed to utilize mRNA vaccine technology targeting conserved antigens (IpaB and IpaC) to overcome the limitations of previous *Shigella* vaccines. By demonstrating functional immune responses against *Shigella flexneri*, our findings suggest that mRNA vaccines may serve as a preventive strategy against *Shigella* infection and contribute to the foundation for future research.

## INTRODUCTION

Shigellosis is an acute diarrheal disease caused by *Shigella* spp., a genus of Gram-negative, non-motile, and facultative intracellular bacteria (1). The disease ranges from mild watery diarrhea to severe inflammatory or bloody diarrhea, accompanied by abdominal pain, fever, and dehydration. Transmission is predominantly through the fecal-oral route, often via contaminated food or water (2). The global burden of shigellosis is particularly severe in resource-limited settings where access to clean water and proper sanitation is inadequate. According to global health estimates, approximately 200,000 deaths annually can be attributed to shigellosis, with the majority of cases occurring in low- and middle-income countries (LMICs) (3). Furthermore, the increasing number of cases among travelers and military personnel stationed in these areas threatens global health security (4). Shigellosis is treatable with antibiotics, but its frequent complications with antimicrobial resistance pose the need for another preventative strategy (5, 6). Despite its significant public health impact, no licensed vaccines are currently available for *Shigella* (7). To reduce the morbidity and mortality associated with shigellosis, it is essential to prioritize vaccine development.

The genus *Shigella* includes four species: *Shigella flexneri*, *Shigella dysenteriae*, *Shigella sonnei*, and *Shigella boydii*, comprising more than 50 serotypes: 16 serotypes for *S. flexneri*, 15 serotypes for *S. dysenteriae*, 1 serotype for *S. sonnei*, and 19 serotypes for *S. boydii*. These serotypes are classified based on the composition of the O-polysaccharide (OPS) in their lipopolysaccharide (LPS) (8). This antigenic diversity causes difficulties in vaccine development, as serotype-specific immunity offers limited cross-protection (9). Therefore, an ideal *Shigella* vaccine should provide broad protection across multiple serotypes, overcoming the serotype-specific limitations of many current vaccine candidates.

To date, various vaccine candidates have been evaluated for *Shigella*, including the use of orally delivered live-attenuated (10–12) or killed strains (13, 14), OPS-based conjugates (15, 16), and LPS-protein mixtures (17, 18). While these candidates have shown potential, they are associated with significant limitations. Many were found to be poorly immunogenic or excessively reactogenic in clinical trials. Moreover, their reliance on OPS specificity has restricted their ability to provide broad-spectrum protection (19). One promising alternative is targeting highly conserved proteins to achieve broad-spectrum protection against various serotypes of *Shigella*. Among protein-based vaccine candidates, components of the Type III Secretion System (T3SS) have shown potential (20–23).

T3SS, encoded on a 220 kb virulence plasmid, is the most important pathogenicity mechanism of *Shigella* species. This needle-like apparatus, embedded in the bacterial cell wall, enables the bacteria to invade host epithelial cells and evade immune responses (24). Among the key components of the T3SS are the invasion plasmid antigen (Ipa) proteins, IpaB and IpaC, which play essential roles in forming the needle tip and translocator. These proteins are highly conserved across all *Shigella* serotypes, making them promising targets for vaccine development (25, 26). Importantly, *Shigella* infections elicit a robust immune response against Ipa proteins, with a significant proportion of infected individuals generating strong responses to IpaB and IpaC (20, 27, 28). This dominant immune response emphasizes the immunogenicity of Ipa proteins and their critical role in host–pathogen interactions. Indeed, Invaplex, a macromolecule subunit vaccine containing IpaB and IpaC proteins along with LPS elicited high levels of protective efficacy against different serotypes of *Shigella* (*S. flexneri* serotype 2a and *S. sonnei*) in preclinical studies (17, 29). Therefore, vaccines targeting IpaB and IpaC are promising approaches for providing broad-spectrum protection against *Shigella*.

mRNA vaccines have emerged as a transformative platform in recent years, with their efficacy and efficiency demonstrated during the COVID-19 pandemic (30). A key advantage of mRNA vaccines is their rapid design and manufacturing process, which allows for adaptable targeting of antigenically diverse pathogens like *Shigella* (31). Furthermore, unlike conventional vaccines, mRNA vaccine formulations enhance vaccine efficacy without additional adjuvants (32, 33). With these advantages, mRNA vaccines could address multiple serotypes and overcome the limitations of protein-based vaccines, which exhibit relatively low immunogenicity in the absence of immune-enhancing strategies such as adjuvants. In this study, we designed two mRNA vaccine constructs targeting the Ipa proteins (IpaB and IpaC), which are highly conserved antigens across *Shigella* species (Fig. S1), to enhance their immunogenicity and protective efficacy. Using a mouse *Shigella* infection model, we evaluated the humoral immune response and the protection capacity provided by the vaccines against *Shigella*.

## MATERIALS AND METHODS

### Bacterial strains characterization

The *Shigella* strains used in this study*—S. flexneri* (serotype 2a, NCCP 14744) and *S. dysenteriae* (serotype 12, NCCP 16190)—were purchased from the National Culture Collection for Pathogens (NCCP, Cheongju, Korea). The strains were streaked and grown on tryptic soy agar (TSA) containing 0.01% Congo Red at 37°C. After 24 hours of incubation, a red colony was selected and subcultured (Fig. S2). *E. coli* DH5α strain (Enzynomics, Daejeon, Korea) was grown overnight in Luria-Bertani (LB) agar at 37°C. A single colony was picked and grown overnight in tryptic soy broth (TSB) or LB broth at 37°C with agitation at 200 rpm. Then, 0.1% of the precultured bacterial suspension was reinoculated in fresh media and grown to an OD_600_ of 0.8–0.9. The cultures were centrifuged, and the pellets were resuspended in PBS or TSB. CFU was determined by plating serial dilutions of bacterial suspension on TSA or LB agar plates.

For PCR, bacterial DNA was extracted using the boiling method. Briefly, bacterial suspensions were centrifuged at 18,400 × *g*, and the pellet was resuspended in distilled water (DW). The suspension was then boiled at 95°C for 15 minutes, cooled on ice, and centrifuged at 18,400 × *g* for 5 minutes. The supernatant containing the bacterial DNA was collected and used as the PCR template. PCR was performed to detect a 606 bp fragment of the *ipgC* gene, located on the virulence plasmid of *Shigella*. The primers used were as follows: forward (5′-GCTATAGCAGTGACATGG-3′) and reverse (5′-ACGAGTTCGAAGCACTC-3′).

Protein sequences of IpaB and IpaC from the two strains were aligned and visualized using BioEdit software (available at https://bioedit.software.informer.com).

### Design of mRNA constructs

Two types of mRNA constructs (total four) were designed to encode the *Shigella* IpaB and IpaC antigens. Each construct contained either a non-optimized or codon-optimized coding sequence (CDS) for the respective antigen. Non-optimized versions (IpaB/C-WT) of sequences for *ipaB* and *ipaC* were derived directly from *Shigella flexneri* NCCP 14744. Codon-optimized versions (IpaB/C) were generated using the codon optimization tool provided by VectorBuilder (https://en.vectorbuilder.com/tool/codon-optimization.html). The sequences were optimized for the murine translation system based on *ipaB* and *ipaC* gene sequences retrieved from the NCBI database (gene accession number NC_004851.1). The identity percentage between the wild-type and codon-optimized DNA sequences was approximately 72.2% for *ipaB* and 69.5% for *ipaC*.

The CDS were cloned into the CUK3-1 plasmid vector (kindly provided by the Catholic University of Korea), which contains essential backbone elements, including a T7 promoter, 5′- and 3′-untranslated regions (UTRs), and a 100-nucleotide poly-A tail. A linker sequence (A50LA50, 20 nucleotides) was incorporated within the poly-A tail, interrupting it to improve mRNA stability and translational efficiency.

### *In vitro* transcription and purification of mRNA

The plasmid CUK3-1 containing the CDS was linearized and purified prior to *in vitro* transcription (IVT). IVT was performed using the EZ T7 High Yield *In Vitro* Transcription Kit (Enzynomics, Daejeon, Korea) according to the manufacturer’s protocol. The reaction included a Cap 1 capping analog (SMARTCAP, ST PHARM, Seoul, Korea) and N1-methylpseudouridine-5′-triphosphate (m1ΨTP) (TriLink, CA, USA) as a substitute for uridine-5′-triphosphate (UTP) to enhance mRNA stability and reduce immunogenicity. The synthesized mRNA was purified using a lithium chloride (LiCl) precipitation method, followed by cellulose-based purification to eliminate double-stranded RNA (dsRNA). The integrity and purity of the mRNA were assessed by agarose gel electrophoresis under denaturing conditions, and its concentration was quantified spectrophotometrically.

### LNP preparation and characterization

Lipid nanoparticles (LNPs) were prepared following a previously reported protocol (34). Briefly, the lipid composition consisted of SM-102 (ionizable lipids), DSPC (1,2-distearoyl-sn-glycero-3-phosphocholine), cholesterol, and 1,2-dimyristoyl-rac-glycero-3-methoxypolyethylene glycol-2000 (DMG-PEG2000) in a molar ratio of 50:10:38.5:1.5. The mRNA and lipid components were mixed in a 50 mM sodium citrate buffer (pH 4.0). LNPs were synthesized using the enCELL-Master V2 system (ENPARTICLE, Busan, Korea) by combining the aqueous and organic phases at a 3:1 ratio with a total flow rate of 10 mL/min. The formulated LNPs were then concentrated via ultrafiltration with an Amicon Ultra centrifugal filter (UFC9030, Merck Millipore, MA, USA), followed by buffer exchange into DPBS. Particle size and polydispersity index (PDI) were measured using dynamic light scattering (DLS).

### Cell transfection and Western blotting

TC-1 cells (generously provided by Seoul National University Hospital, Korea) were cultured in DMEM supplemented with 10% fetal bovine serum (FBS) and 1% penicillin-streptomycin. For transfection, cells were seeded at 70–80% confluency and treated with 4 µg of mRNA delivered using either Lipofectamine 2000 (Invitrogen, Waltham, MA, USA) or LNP. After 24 hours, cells were lysed in RIPA buffer containing protease inhibitors, and protein concentrations were determined using a BCA assay. Proteins were separated on 10% SDS-PAGE gels, transferred to nitrocellulose membranes, and blocked with 5% skim milk in TBST for 1 hour. The membranes were incubated overnight at 4°C with primary antibodies specific to IpaB and IpaC (1:2,000 dilution, CusaBio, Houston, TX, USA). Subsequently, HRP-conjugated secondary antibodies (1:50,000 dilution, SouthernBiotech, Birmingham, AL, USA) were incubated with the membrane for 1 hour. Protein bands were visualized using an ECL substrate solution (BIOMAX, Guri, Korea).

### Mice, immunizations, and infection

C57BL/6 female mice (6 weeks old) were purchased from Orient Bio Inc. (Sungnam, Korea) and housed under specific pathogen-free conditions at the Lee Gil Ya Cancer and Diabetes Institute (Incheon, Korea).

For the determination of the optimal doses of *S. flexneri* and *S. dysenteriae*, the bacteria were prepared at concentrations of 5 × 10⁷, 1 × 10⁷, and 5 × 10⁶ CFU and were used to infect mice intraperitoneally. Survival rates and body weight changes were monitored for up to 4 days post-infection (dpi).

For evaluation of vaccine efficacy, mice were divided into groups (*n* = 3–6 per group) and immunized intramuscularly on days 0, 14, and 28 with LNP-encapsulated mRNA vaccines encoding IpaB or IpaC at doses of 5  µg or 20  µg (mRNA content). Control groups received LNPs encapsulating firefly luciferase mRNA. Serum samples were collected biweekly by retro-orbital bleeding under Isoflurane anesthesia with oxygen. On day 42, *S. flexneri* was incubated for 4 hours, centrifuged, and resuspended in PBS to a final concentration of 1 × 10⁷ CFU. Mice were challenged intraperitoneally on day 42. Survival rates and body weight changes were monitored for up to 7 dpi. At the end of the experiments, mice were euthanized by CO₂ inhalation.

### Fecal CFU determination

Fecal samples were collected from mice at 28 hours post-infection (hpi) and homogenized in PBS (100 µg/100 µL). Serial dilutions were plated on TSA containing ampicillin and 0.01% Congo Red to select for *Shigella* colonies, as the strains used in this study are intrinsically resistant to ampicillin (35). Plates were incubated at 37°C for 24 hours.

### Histology

At 28 hours post-infection (hpi), mice were euthanized, and colon tissues were harvested. The colon length was measured. Then, the tissues were cut longitudinally to remove luminal contents, swiss-rolled, and fixed in 10% neutral-buffered formalin. Subsequently, samples were embedded in paraffin. Sections (5 µm thick) were stained with hematoxylin and eosin (H&E) for histological analysis. Pathological changes were evaluated under a light microscope. Paraffin embedding, sectioning, H&E staining, and imaging were conducted by the Central of Animal Care Use (CACU). The histological analyses were done by using instruments at the Core Facility for Cell-to-In Vivo Imaging (Incheon, Korea).

### Anti-IpaB, IpaC enzyme-linked immunosorbent assay (ELISA)

Anti-IpaB and IpaC-specific IgG levels in serum samples were quantified using an indirect enzyme-linked immunosorbent assay (ELISA). The blank ELISA plates (Greiner Bio-One, Kremsmünster, Austria) were coated with 2 µg/well of IpaB or 200 ng/well of IpaC proteins (CusaBio, Houston, TX, USA) and incubated overnight at 4°C with gentle shaking. After washing four times with PBST, 5% skim milk was added to block each well and blocked at room temperature (RT) for 2 hours. After two washes, serum samples were diluted 1:50 in 5% skim milk and were added to the wells. Plates were incubated at 37°C for 2 hours. Antibody binding was detected using HRP-conjugated secondary antibodies (1:2,000 dilution, SouthernBiotech, Birmingham, AL, USA) and incubated at 37°C for 1 hour. Then, 100 µL of TMB substrate solution (GenDepot, Barker, TX, USA) was added to each well and incubated for 15 minutes in the dark. The reaction was stopped by adding 100 µL of stop solution, and absorbance was measured at 450 nm.

### Invasion assay

To evaluate the ability of *S. flexneri* and *S. dysenteriae* to invade host cells, an invasion assay was conducted. Caco-2 cells, purchased from the Korea Cell Line Bank (KCLB), were seeded as a single layer in 6-well plates. Each *Shigella* strain, along with *E. coli* DH5α used as a control, was used to infect the cells at a multiplicity of infection (MOI) of 5, followed by incubation at 37°C for 2 hours. The mixture was then added to the cells and incubated for 2 hours at 37°C. After the incubation, cells were washed twice, and media containing gentamicin was added and incubated for 1 hour at 37°C to remove extracellular bacteria that had not invaded the cells. The cells were then washed twice more, lysed, and the number of intracellular bacteria quantified by serial dilution and plating on agar.

To assess the inhibitory effects of vaccine-induced antibodies on *Shigella* invasion, an invasion inhibition assay was conducted. Caco-2 cells were seeded as a single layer in 96-well plates. Heat-inactivated serum samples were pre-incubated with *S. flexneri* or *S. dysenteriae* at an MOI of 5 for 30 minutes at 37°C. The following procedure was identical to the invasion assay described above. Invasion inhibition was calculated relative to the control groups.

### Graph schematics

IVT vector and vaccination schematic illustrations were created by Biorender.com.

### Statistical analysis

All statistical analyses were performed using GraphPad Prism 10 statistical software. Comparisons between two groups were conducted using Student’s *t*-test. For comparisons between multiple groups, one-way ANOVA or two-way ANOVA with Dunnett’s post-hoc test was used. Survival analysis was performed using the log-rank (Mantel–Cox) test. A *P*-value of <0.05 was considered statistically significant (ns = not significant, *P < 0.05, **P < 0.01, ***P < 0.001, and ****P < 0.0001 compared to control). Data are presented as mean ± standard deviation (SD) or mean ± standard error of the mean (SEM). These details are provided in the figure legends.

## RESULTS

### Construction of mRNA vaccine encoding *Shigella* IpaB and IpaC proteins

**Fig 1:**
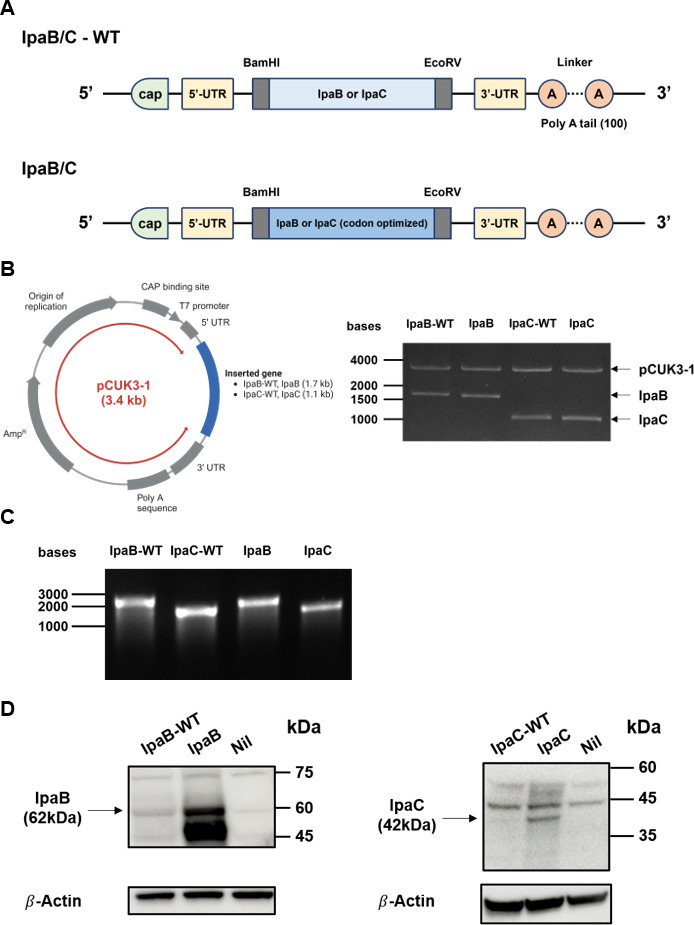
Construction of mRNA vaccine. (**A**) Schematic illustration of mRNA constructs used in this study. The mRNA constructs consist of 5′ cap followed by 5′ UTR, IpaB or IpaC, 3′ UTR, and poly A tail. Both constructs include IpaB or IpaC sequences from *Shigella flexneri* serotype 2a. The construct of IpaB/C-WT contains the original sequences, while the construct of IpaB/C contains codon-optimized versions. (**B**) Characterization of vector for IVT (left panel). Confirmation of IpaB and IpaC sequence insertion into the pCUK3-1 vector by restriction enzyme digestion (right panel). The data show two bands in each lane, corresponding to the vector (pCUK3-1; 3,362 bp) and the inserted IpaB (1,743 bp) or IpaC (1,092 bp) sequences. (**C**) Verification of integrity and purity of IVT products using denaturing agarose gel electrophoresis. A single band for either mRNA-IpaB-WT, IpaB (2,151 bp) or mRNA-IpaC-WT, IpaC (1,500 bp) is observed in each lane. (**D**) Comparison of the *in vitro* expression efficiency of antigen protein with and without codon optimization. Western blot analysis of (**A**) IpaB and (**B**) IpaC expression in cell lysates 24 hours post-transfection with mRNA-IpaB/C-WT or mRNA-IpaB/C. Non-transfected cell lysates were included as a negative control. β-actin was used as a loading control. WT, wild type; 5′ UTR, 5′ untranslated region; 3′ UTR, 3′ untranslated region; poly A tail, polyadenylic tail; IVT, *in vitro* transcription.

We designed two types of constructs to develop an mRNA vaccine using *Shigella* Ipa proteins as antigens (Fig. 1A). The first construct, named IpaB/C-Wild Type (WT), includes either the original IpaB or IpaC sequence from *S. flexneri* serotype 2a inserted between the 5′-UTR and 3′-UTR. The second construct, named IpaB/C, contains a codon-optimized version of the corresponding sequence adjusted for the mouse translation system. We hypothesized that the codon-optimized IpaB/C construct would result in higher levels of protein expression in mouse cells compared to the WT construct. We then sought to test this hypothesis through experimental comparison. To generate the constructs, either codon-optimized or non-optimized IpaB and IpaC sequences were cloned into the *in vitro* transcription (IVT) vector (plasmid CUK3-1) (Fig. 1B). The cloned plasmids were linearized and used for IVT to synthesize mRNA. The gel electrophoresis results of the IVT products showed bands corresponding to the expected sizes of each construct (Fig. 1C). These results confirmed the successful synthesis of mRNA for the constructs. To compare the impact of codon optimization on protein expression efficiency, the mRNA constructs were transfected into the mouse cell line TC-1, and protein expression levels were assessed (Fig. 1D). As shown in Fig. 1D, more intense bands for both IpaB and IpaC are detected in the lanes of the codon-optimized constructs. These results suggest that codon optimization for mouse expression significantly increased the translation efficiency of the mRNA in the TC-1 mouse cell line. Therefore, the IpaB/C construct with higher protein expression was used in subsequent experiments.

### Formulation and characterization of mRNA-IpaB-LNP and mRNA-IpaC-LNP

**Fig 2:**
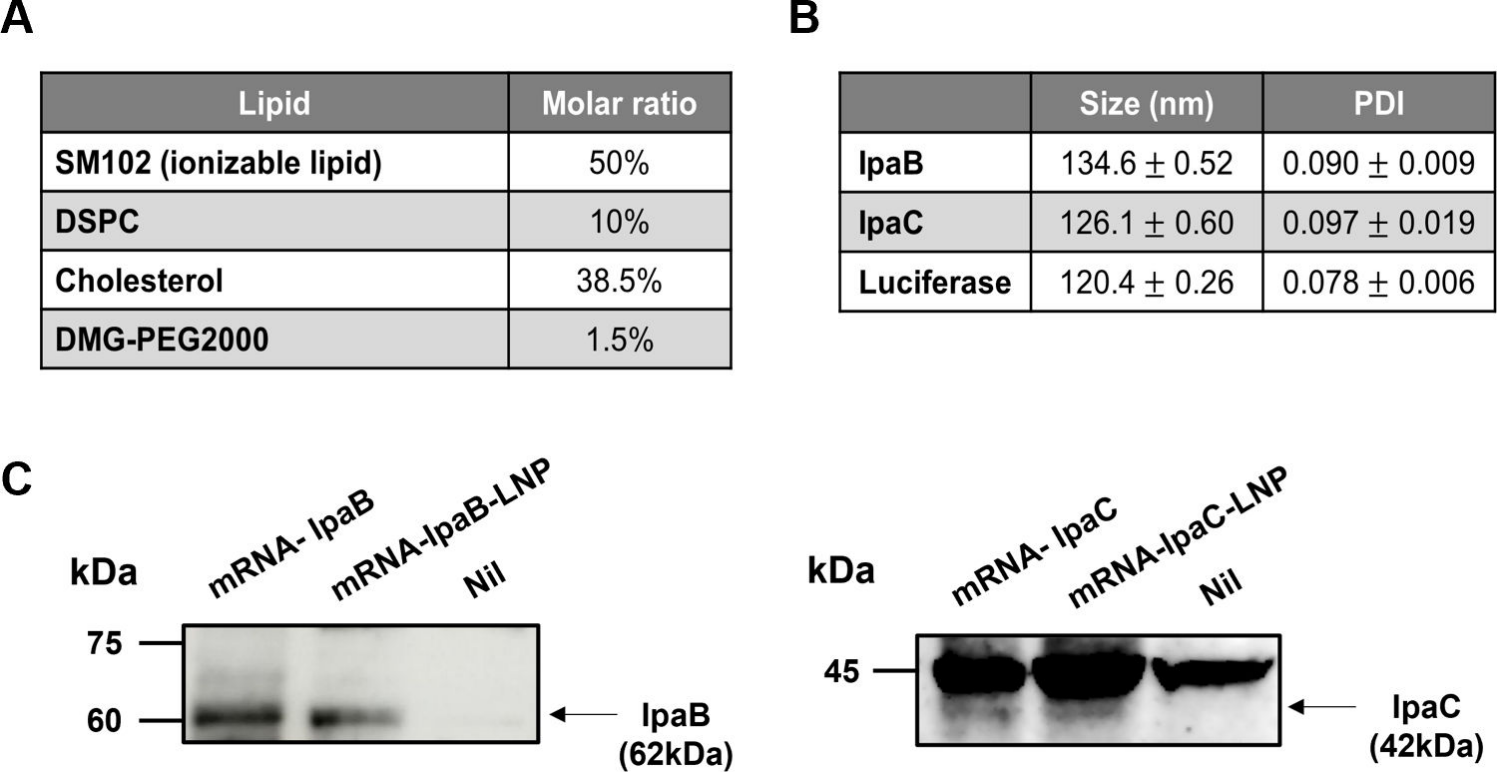
Characterization of LNPs. (**A**) LNP formulation for mRNA delivery used in this study. (**B**) Physicochemical characterizations of mRNA-LNP used in this study. Particle size and PDI of mRNA-IpaB/C-LNP were measured by dynamic light scattering (DLS). mRNA-firefly luciferase-LNP was included as a negative control. (**C**) Verification of antigen expression through mRNA-IpaB/C-LNP. Western blot analysis of (**A**) IpaB and (**B**) IpaC expression in cell lysates 24 hours post-transfection with mRNA-IpaB/C-LNP. Cell lysates transfected with mRNA-IpaB/C were used as a positive control, while non-transfected cell lysates were used as a negative control. PDI, polydispersity index.

To ensure stable delivery of the synthesized mRNA into cells, lipid nanoparticles (LNPs) were utilized. The composition of the LNPs is presented in Fig. 2A. For quality control, the particle size and polydispersity index (PDI) of mRNA-IpaB and mRNA-IpaC encapsulated in LNPs are measured and summarized in Fig. 2B. The average size of the particles resulted in 134.6 nm and 126.1 nm, respectively. To confirm whether the LNPs effectively deliver the mRNA into cells, mRNA-IpaB-LNP and mRNA-IpaC-LNP were treated with TC-1 cells, and protein expression levels were evaluated (Fig. 2C). As shown in Fig. 2C, the vaccine candidates mRNA-IpaB-LNP and mRNA-IpaC-LNP successfully deliver to the mouse cell line, resulting in effective expression of the target antigens within the cells.

### Design of *Shigella* challenge system for vaccine evaluation

**Fig 3:**
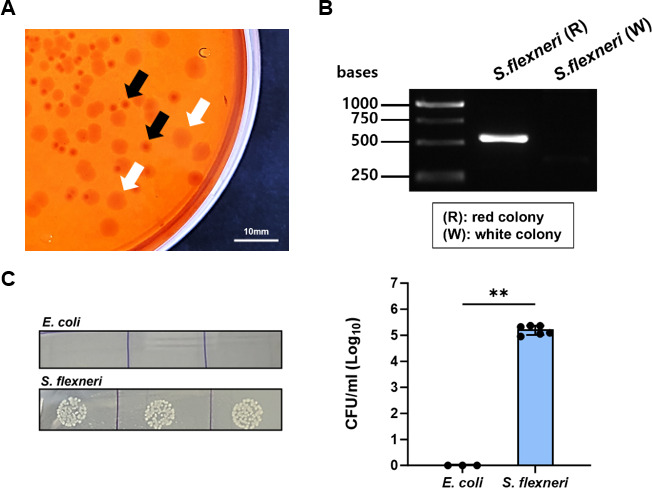
Evaluation of pathogenicity in *S. flexneri* strain. (**A**) Close-up view of *S. flexneri* colonies on CR agar showing two distinct colony morphologies. Black arrows indicate red, virulent colonies (R), and white arrows indicate white, non-virulent colonies (W). Scale bar represents 10 mm. (**B**) Detection of plasmid gene within the strains by PCR. A band corresponding to a 606 bp fragment of the virulence plasmid gene *IpgC* was detected in the *S. flexneri* (R) lane. (**C**) Confirmation of invasion ability in *Shigella* strains. Lysates from cells infected with *S. flexneri* (R) were diluted and plated in triplicate on agar plates (left panel). The number of colonies was quantified and presented as a graph (right panel). Each symbol represents one biological replicate (*n* = 3–6). Two-tailed Student’s *t*-test was performed (**P < 0.01). Data are presented as mean ± SEM. R, red colony; W, white colony.

As part of establishing a *Shigella* challenge system for vaccine evaluation, we needed to obtain virulent *Shigella* strains and optimized a mouse infection model to suit their characteristics. Initially, we acquired two serotypes of *Shigella* strains from the National Culture Collection for Pathogens (NCCP): *S. flexneri* (serotype 2a) and *S. dysenteriae* (serotype 12) (Fig. 3A; Fig. S3A). *Shigella* strains are known to frequently lose their virulence plasmid under laboratory conditions. Strains lacking the plasmid exhibit impaired invasion capabilities, leading to a reduction in virulence (35). To select for virulent strains, we examined the colony morphology of the strains on Congo Red (CR) agar. In general, relatively small and uniform red colonies on CR agar are known to indicate the presence of the virulence plasmid, while relatively large and irregular white colonies are associated with the absence of the virulence plasmid (36). Among the strains we obtained, *S. flexneri* exhibited both red and white colony forms on CR agar (Fig. 3A). On the other hand, *S. dysenteriae* formed only red colonies (Fig. 3A). We also verified the presence of the virulence plasmid within these strains by PCR (Fig. 3B; Fig. S3B). PCR results showed that the virulence plasmid gene was detected in the red colonies of *S. flexneri* and in *S. dysenteriae*, consistent with the observed colony morphology. The virulence of these two strains was confirmed through a gentamicin protection assay. Both strains were used to infect the human colorectal cancer cell line Caco-2 under *in vitro* conditions. The results showed a higher invasion ability compared to the *E. coli* DH5α strain, which does not exhibit invasion capability (Fig. 3C; Fig. S3C). Therefore, the virulence of both *S. flexneri* and *S. dysenteriae* was confirmed, and these virulent strains were used for further vaccine efficacy evaluation in subsequent experiments.

**Fig 4:**
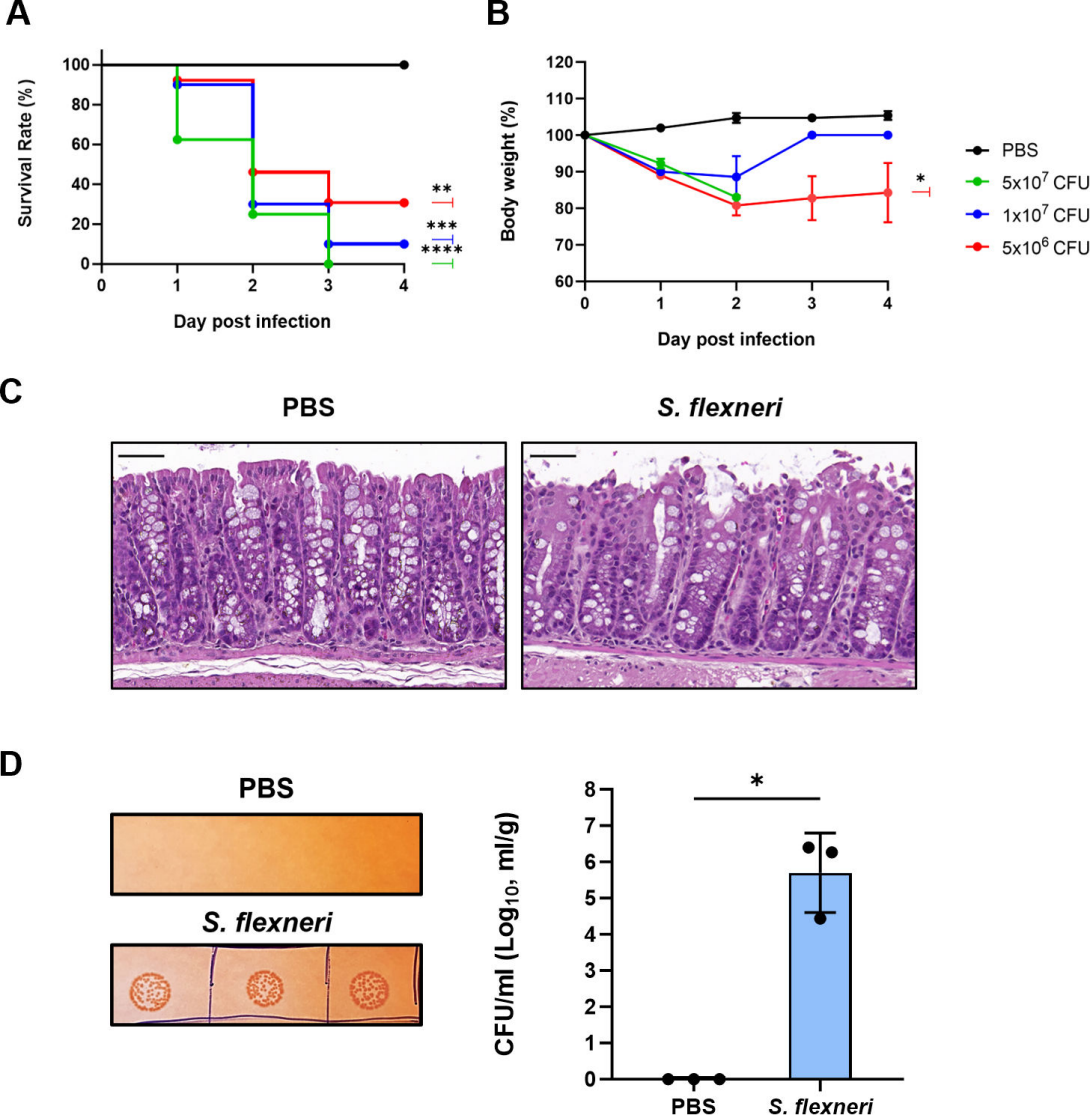
*In vivo* pathogenicity assessment of *S. flexneri* strain. Adult mice were injected via the intraperitoneal (IP) route with three doses (5 × 10^6^, 1 × 10^7^, or 5 × 10^7^ CFU) of *Shigella* strains (8–13 mice per group). Survival rates (**A**) and group-wise mean body weight changes (**B**) were monitored up to 4 dpi. (**C**) Images of H&E-stained colon tissue from infected mice. Scale bar represents 50 µm. (**D**) Confirmation of *Shigella* colonization in the mouse mucosal system. Homogenates of feces from infected mice were diluted and plated in triplicate on agar plates (left panel). The number of colonies was quantified and presented as a graph (right panel). Each symbol represents two fecal samples from mice (each group; *n* = 3). Mean ± SEM is shown in (**B and D**). Statistical analyses were performed using log-rank (Mantel–Cox) test (**A**), one-way ANOVA with Dunnett’s multiple comparisons test (**B**), and two-tailed Student’s *t*-test (**D**) (ns = not significant, *P < 0.05, **P < 0.01, ***P < 0.001, and ****P < 0.0001 compared to PBS control).

To examine whether *S. flexneri* and *S. dysenteriae* cause pathogenesis under *in vivo* conditions, a mouse intraperitoneal (IP) infection model was used (37). The optimal infection dose was determined by administering three different concentrations of the two strains via the IP route to C57BL/6 mice. Survival rates and body weight changes were monitored up to 4 days post-infection (dpi). In the *S. flexneri* infection group, the highest dose of 5 × 10^7^ CFU resulted in 0% survival rate within three days, while the 1 × 10^7^ CFU dose showed 10% survival rate, and the 5 × 10^6^ CFU dose resulted in approximately 30.8% survival rate (Fig. 4A). In the *S. dysenteriae* infection group, the 5 × 10^7^ CFU dose resulted in 0% survival rate, the 1 × 10^7^ CFU dose showed 30% survival rate, and the 5 × 10^6^ CFU dose resulted in 62.5% survival rate (Fig. S4A). Infected mice exhibited significant weight loss for three days (Fig. 4B; Fig. S4B). To avoid potential masking of vaccine efficacy in subsequent efficacy evaluations due to excessively high infection doses, the optimal infection dose for both strains was determined to be 1 × 10^7^ CFU.

To investigate whether *Shigella* infection induces inflammatory responses in the mouse colon, colons were harvested from mice infected with 1 × 10⁷ CFU of *Shigella* strains at 28 hours post-infection (hpi). Histological analysis of colon tissue stained with H&E revealed epithelial shedding and compromised barrier integrity in mice infected with *Shigella* (Fig. 4C; Fig. S4C). Additionally, a significant number of *Shigella* organisms were detected in the feces of infected mice at 28 hpi (Fig. 4D; Fig. S4D). These findings confirm that IP infection with 1 × 10⁷ CFU of *S. flexneri* or *S. dysenteriae* induces inflammatory responses in the mouse colon and enables colonization of the murine mucosal system.

### Validation of mRNA vaccine’s protective efficacy against *Shigella*

**Fig 5:**
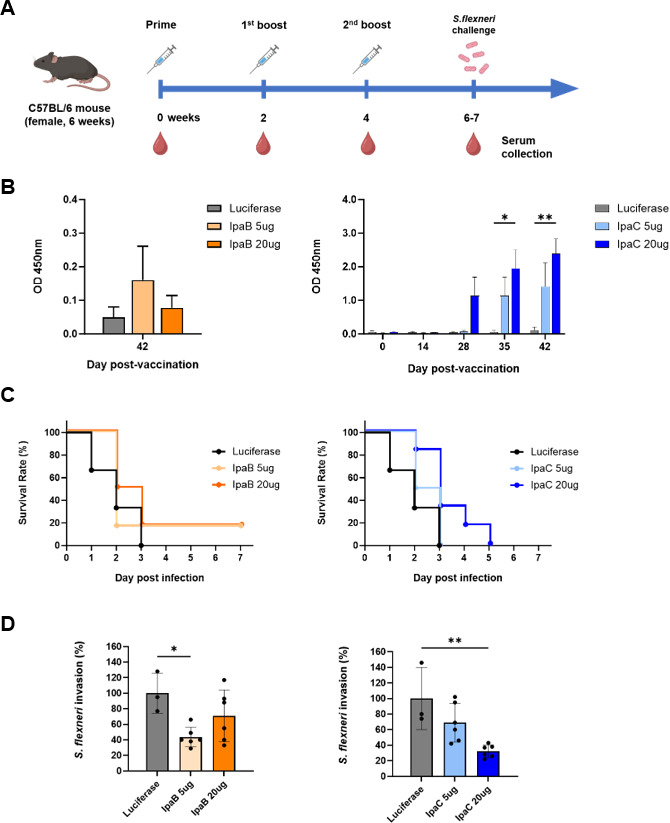
Evaluation of mRNA vaccine efficacy against *S. flexneri*. (**A**) Schematic diagram of immunization, sample collection, and challenge. Adult mice were vaccinated intramuscularly (IM) on days 0, 14, and 28 with 5 µg or 20 µg of mRNA vaccines (*n* = 6/group). A group vaccinated with mRNA-luciferase-LNP (luciferase group) was included as a negative control (*n* = 3/group). Blood samples were collected every two weeks. On days 42 or 49, all groups of mice were challenged with 1 × 10^7^ CFU of *S. flexneri*. (**B**) Anti-IpaB IgG (left panel) and anti-IpaC IgG (right panel) levels in immunized serum measured by ELISA. (**C**) Survival curves of mice vaccinated with three doses of mRNA-IpaB vaccine (left panel) or mRNA-IpaC vaccine (right panel). (**D**) *S. flexneri* invasion (percentage of CFU) into Caco-2 cells after treatment with immunized serum (42 days post-vaccination). The number of invaded bacteria treated with serum of the luciferase group was set to 100%. Each symbol represents one mouse (*n* = 3–6). Data are representative of one independent experiment. Mean ± SEM is shown in (**B**), and mean ± SD is shown in (**D**). Statistical analyses were performed using one-way ANOVA or two-way ANOVA with Dunnett’s multiple comparisons test (**B and D**). Log-rank (Mantel–Cox) test was used for survival plot (**C**) (ns = not significant, *P < 0.05, **P < 0.01, and ***P < 0.001 compared to luciferase group as a control).

To evaluate whether our vaccines provide protection against *Shigella*, we used the previously established mouse infection model. Mice were immunized intramuscularly (IM) with IpaB and IpaC vaccines at doses of 5 µg (low dose) or 20 µg (high dose). The control group received a formulation encoding firefly luciferase. Vaccinations were administered three times at two-week intervals. Two or three weeks after the second boost, mice were challenged with *S. flexneri*, and survival rates were monitored for 7 dpi. The experimental scheme is shown in Fig. 5A.

Serum samples were collected at two-week intervals throughout the vaccination period. To assess whether the vaccines induced antigen-specific antibodies, IgG ELISA was performed on the collected serum samples (Fig. 5B). As shown in the left panel of Fig. 5B, two weeks after the second boost, the levels of IpaB-specific IgG are not statistically significant, but the 5 µg group exhibits a slight trend of higher levels compared to the 20 µg group. In contrast, serum samples from mice immunized with the IpaC vaccine showed a dose-dependent increase in IgG levels (Fig. 5B, right panel). Additionally, a time-dependent anti-IpaC humoral response was observed. These results confirm that both IpaB and IpaC vaccines can induce humoral anti-IpaB or anti-IpaC responses.

Next, the protective efficacy of the vaccines against *Shigella* was evaluated by challenging *S. flexneri* in vaccinated mice with either the mRNA vaccine or mRNA-firefly luciferase-LNP as a control (Fig. 5C). In the groups vaccinated with the IpaB vaccine, both the 5 µg and 20 µg doses showed approximately 17% survival rates. For the IpaC vaccine, although none of the groups survived the full seven-day observation period, survival rates during the first 5 dpi exhibited a dose-dependent trend.

The challenge experiment revealed that vaccinated groups survived slightly longer than unvaccinated controls. Previous studies suggest that neutralizing antibodies against Ipa proteins play a critical role in protection against *Shigella* (9, 38–40). Based on these findings, we hypothesized that our vaccines successfully induced neutralizing antibodies, which contributed to the extended survival observed in the vaccinated groups. To investigate this hypothesis, an invasion inhibition assay using heat-inactivated serum was conducted to assess whether our vaccines induced neutralizing antibodies inhibiting *Shigella* invasion into host cells (Fig. 5D). When the invasion level of the control was set to 100%, the IpaB vaccine group exhibited an invasion level of approximately 49% in the 5  µg dose group and 72% in the 20  µg dose group. The IpaC vaccine group showed an invasion level of approximately 61% in the 5  µg dose group and 43% in the 20  µg dose group. These results confirm that both vaccines can induce neutralizing antibodies. Furthermore, the observation that groups with higher bacterial invasion inhibition showed prolonged survival suggests that greater neutralizing antibody capacity was induced in these groups. This highlights the critical role of neutralizing antibodies in providing protection against *Shigella*.

**Fig 6:**
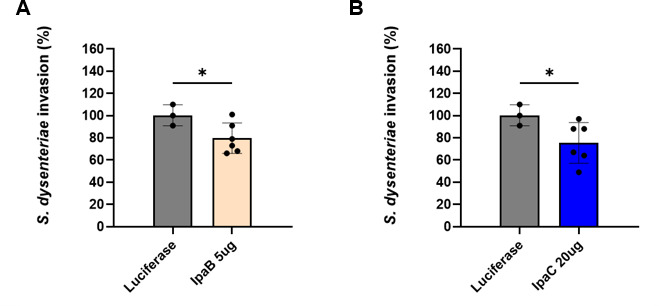
Evaluation of invasion inhibition of *S. dysenteriae* by mRNA vaccine-derived antibodies. Percentage of *S. dysenteriae* invasion (measured as CFU) into Caco-2 cells after treatment with serum from mice immunized with mRNA-IpaB vaccine (**A**) and mRNA-IpaC vaccine (**B**) at 42 days post-vaccination. The number of invaded bacteria treated with serum of the luciferase group was set to 100%. Each symbol represents one mouse (*n* = 3–6). Two-tailed Student’s *t*-test was performed (*P < 0.05). Data are representative of one independent experiment. Data are presented as mean ± SD.

Since our vaccines target conserved proteins within *Shigella* serotypes, we anticipated that our vaccines could provide cross-protection against serotypes other than *S. flexneri*. To confirm this, we performed an invasion inhibition assay using *S. dysenteriae* (Fig. 6). The immunized sera from the IpaB 5 µg group and the IpaC 20 µg group, which showed higher inhibition rates against *S. flexneri* invasion, were used for the assay. Similarly, when the invasion level of the control was set to 100%, the IpaB vaccine group exhibited an inhibition rate of approximately 17%, and the IpaC vaccine group showed an inhibition rate of approximately 21%. These results suggest that the neutralizing antibodies induced by our vaccine also tend to inhibit the invasion of serotypes other than *S. flexneri*.

## DISCUSSION

*Shigella* species are the leading cause of bacterial diarrheal deaths, a significant global health burden. However, despite their importance, an affordable vaccine for *Shigella* has not yet been developed due to the serological diversity. The main limitation of current OPS-based vaccine candidates is their restricted coverage of serotypes. Moreover, conserved protein-based vaccine candidates have generally faced challenges in eliciting sufficient immunogenicity. To overcome these limitations, this study developed mRNA vaccines using the highly conserved Ipa proteins as antigens to address various *Shigella* serotypes.

In our Western blot experiments to verify the intracellular expression of IpaB and IpaC proteins, multiple bands are detected that did not correspond to the expected sizes of the target proteins, as shown in Fig. 1 and 2. Notably, some of these bands also appeared in the negative control lane, suggesting that non-specific binding due to the low target protein binding affinity of the antibodies used may be responsible. For these experiments, we utilized commercial anti-*Shigella* IpaB and IpaC polyclonal antibodies as the primary antibodies. Currently, there are limited commercial antibody options available for *Shigella* IpaB and IpaC proteins, likely due to the challenges associated with purifying recombinant IpaB and IpaC proteins (41–43). While acquiring alternative antibodies poses significant challenges, it remains essential to develop improved antibodies to reduce non-specific bands and enhance the reliability of the results. In Fig. 1D, several intense bands below the target band in the IpaB lane are observed. Since these bands were absent in the negative control, it is reasonable to interpret them as resulting from the antibody binding to epitopes on the target protein rather than non-specific binding. The difference in size compared to the target protein may be attributed to degradation during translation in mammalian cell lines or post-translational modifications (PTMs) altering the protein size. Furthermore, due to limitations in obtaining high-quality recombinant proteins of *Shigella*, we were restricted in measuring significant levels of IpaB-specific antibodies in the serum of vaccinated mice, as we had to use large amounts of recombinant protein. This limitation in protein availability meant that we were only able to measure antibody levels at the final time point, preventing us from confirming antibody induction at the early stages of vaccination. Since verifying the early induction of antibodies is crucial for assessing vaccine efficacy, the development of improved recombinant proteins of higher quality will be necessary for future studies.

*Shigella* is a host-restricted bacterium that primarily infects humans (44). Due to this host specificity, establishing animal models for *Shigella* infection has led to significant challenges for researchers. Considering factors such as cost and limited available tools, the number of viable animal models is currently quite restricted. Among these, the mouse intraperitoneal (IP) infection model we employed offers the advantage of enabling relatively easy infection of mice and observation of their symptoms (37). We optimized this model for *S. flexneri* and *S. dysenteriae* to suit our experimental needs. However, the use of this model in our study revealed several limitations. First, intraperitoneal infection of mice with *Shigella* does not replicate the natural infection route and therefore fails to fully mimic the clinical symptoms of human shigellosis. Second, this route primarily induces systemic immune responses and is not generally associated with the induction of mucosal immunity, making it suitable for evaluating mRNA vaccines. However, it may not fully reflect the mucosal immune responses that are critical for protection against enteric pathogens, such as *Shigella*. As a result, the evaluation of mucosal immune responses—known to be associated with protection against the intestinal pathogen *Shigella*—was limited in this model. Last, intraperitoneal infection often leads to systemic infection rather than localized intestinal disease, resulting in rapid mortality due to bacteremia or other systemic complications (45). These characteristics made it challenging to establish an appropriate infection dose and to analyze disease progression in *Shigella*-infected mice during our experiments. To advance the development of *Shigella* vaccines or therapeutics, it is imperative to establish an animal model that closely reproduces human shigellosis while allowing for detailed analysis of pathology.

As shown in Fig. 5C, the vaccinated mouse groups challenged with *S. flexneri* exhibit a slightly higher survival rate than the control group at 7 dpi, but the difference is not statistically significant. This limited protection may be attributed to insufficient antibody induction. In general, effective antibody production in response to mRNA vaccines requires the antigen to be secreted extracellularly or exposed on the cell surface, thereby enhancing interactions with immune cells after the protein is newly synthesized (46). We previously conducted experiments using TC-1 cells to assess whether antigen proteins encoded by the mRNA vaccines are secreted extracellularly. Under our experimental conditions, however, little to no secretion of antigen proteins into the extracellular space was detected (data not shown). This suggests that the antigen proteins synthesized by our vaccine had limited opportunities to interact with immune cells involved in antibody production, leading to insufficient induction of antigen-specific antibodies. To address this problem, incorporating a signal peptide into the vaccine construct could be a potential solution. Signal peptides are short sequences (approximately 15–25 amino acids in length) that play a critical role in guiding newly synthesized proteins to the secretory pathway and facilitating their translocation and secretion outside the cell (47). Numerous studies on mRNA vaccines incorporating signal peptides have demonstrated enhanced secretion of vaccine antigen proteins and improved immune responses as a result (46, 48, 49). Therefore, the addition of a signal peptide to our vaccine construct is expected to enhance its protective efficacy.

Since previous *Shigella* vaccine studies have suggested that neutralizing antibodies likely play a critical role in protective immunity, this study focused on analyzing humoral immunity to evaluate the usability of the vaccine. However, the formulation characteristics of mRNA vaccines are expected to effectively induce T cell immunity as well, which may significantly contribute to vaccine efficacy. Although the absence of T cell immune response analysis represents a limitation of this study, we acknowledge its importance and plan to address it in future studies.

In this study, we established a system for evaluating the efficacy of vaccines to prevent *Shigella* infections. However, as the survival rates induced by our vaccine did not show a marked difference from those of the control group, we were unable to thoroughly assess disease symptoms resulting from *Shigella* infection or directly determine whether the vaccine provides cross-protection against other serogroups, such as *S. dysenteriae*, through challenges. Nonetheless, if further studies address the limitations of the current vaccine, the experimental framework developed in this study may allow for a more effective evaluation of vaccine efficacy.

In our invasion inhibition assay, we observed a difference in the inhibition rates of *S. flexneri* and *S. dysenteriae* invasion by vaccine-induced neutralizing antibodies. In our previous study, *S. flexneri* and *S. dysenteriae* showed distinct levels of virulence in infected animals. Given the moderate efficacy of our vaccine demonstrated in this study, we believe that the difference in vaccine efficacy against the two strains is likely attributable to inherent differences in their virulence.

In conclusion, our study provides valuable insights into the design of effective mRNA vaccines. Incorporating design optimizations, such as the inclusion of a signal peptide to enhance antigen secretion, could improve antibody induction and vaccine efficacy in future refinements. While the results of this study were not as successful as anticipated, they highlight the importance of thorough optimization in advancing toward more effective and practical vaccine development strategies.

## SUPPLEMENTAL MATERIAL

Figures S1 to S4 spectrum.00993-25-s0001.docx Additional experimental details.

ASM does not own the copyrights to Supplemental Material that may be linked to, or accessed through, an article. The authors have granted ASM a non-exclusive, world-wide license to publish the Supplemental Material files. Please contact the corresponding author directly for reuse.
