# Supplementary material for: Neutralizing antibodies induced by IpaB and IpaC mRNA vaccines inhibit Shigella flexneri invasion
Source: Microbiol Spectr. 2025 Sep 2;13(10):e00993-25. doi: 10.1128/spectrum.00993-25 (PMC12502688; doi:10.1128/spectrum.00993-25)
Supplement: Figures S1 to S4 — Additional experimental details. [file spectrum.00993-25-s0001.docx]

**Supplemental figures**


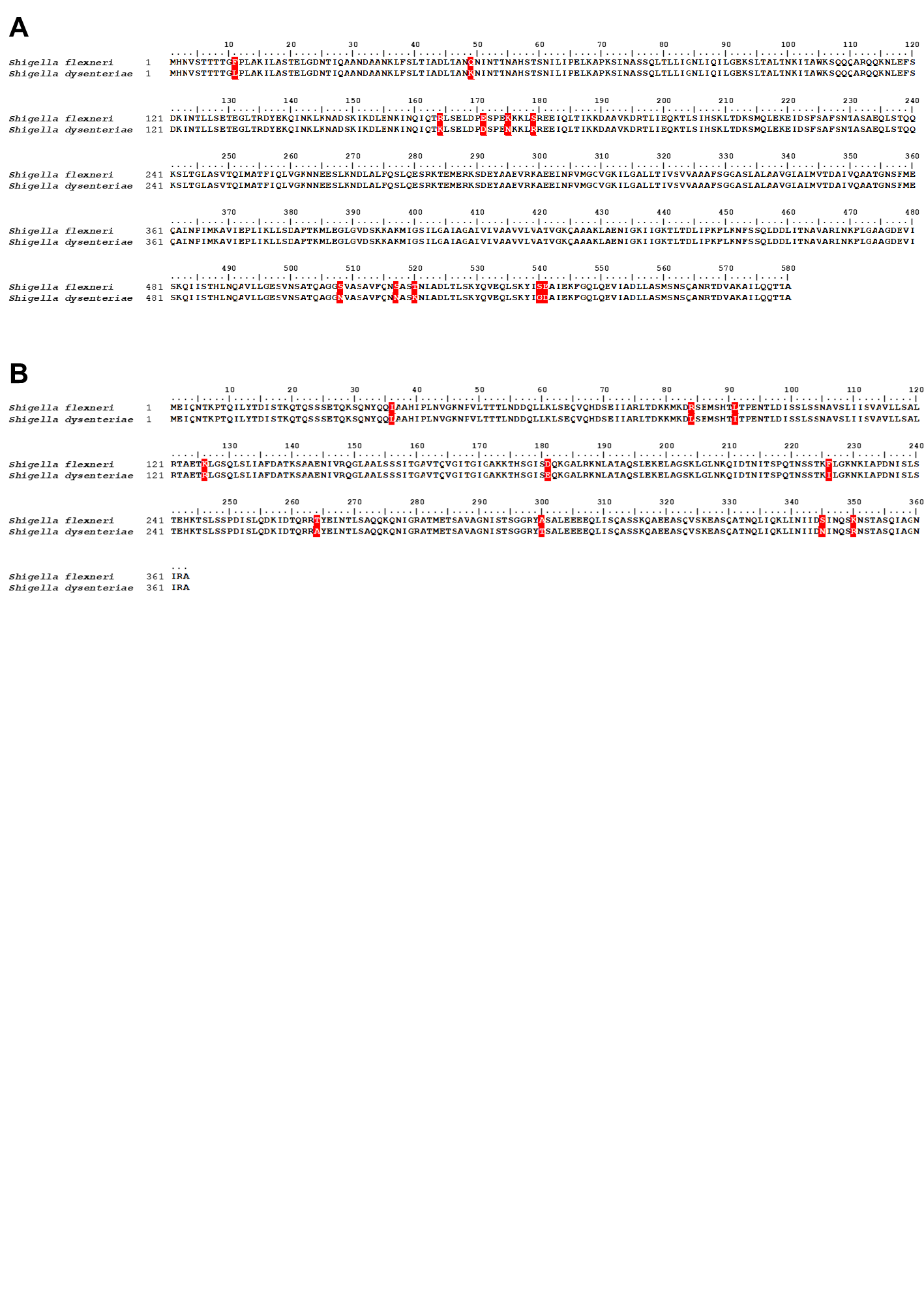


**Supple. Fig. 1.** **Amino acid sequence alignment of IpaB and IpaC proteins from two *Shigella* strains.** Protein sequence alignment of IpaB **(A)** and IpaC **(B)** from *S. flexneri* (NCCP 14744) and *S. dysenteriae* (NCCP 16190) performed using BioEdit software. Red shading indicates sequence variation between the two strains. The sequence identity was 98.1% for IpaB and 97.3% for IpaC.

**
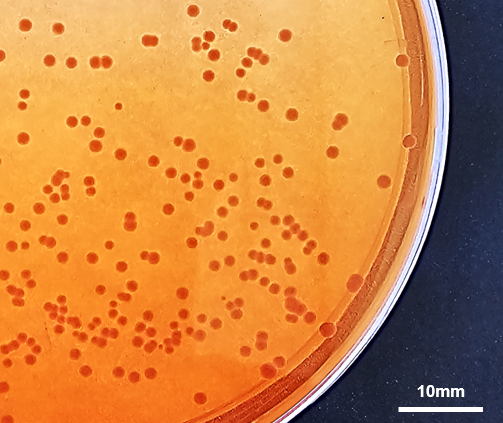
**

Supple. Fig. 2. Colony morphology of *S. flexneri* showing maintenance of red colonies. Close-up view of *S. flexneri* colonies on Conge Red (CR) agar. Scale bar represents 10 mm.


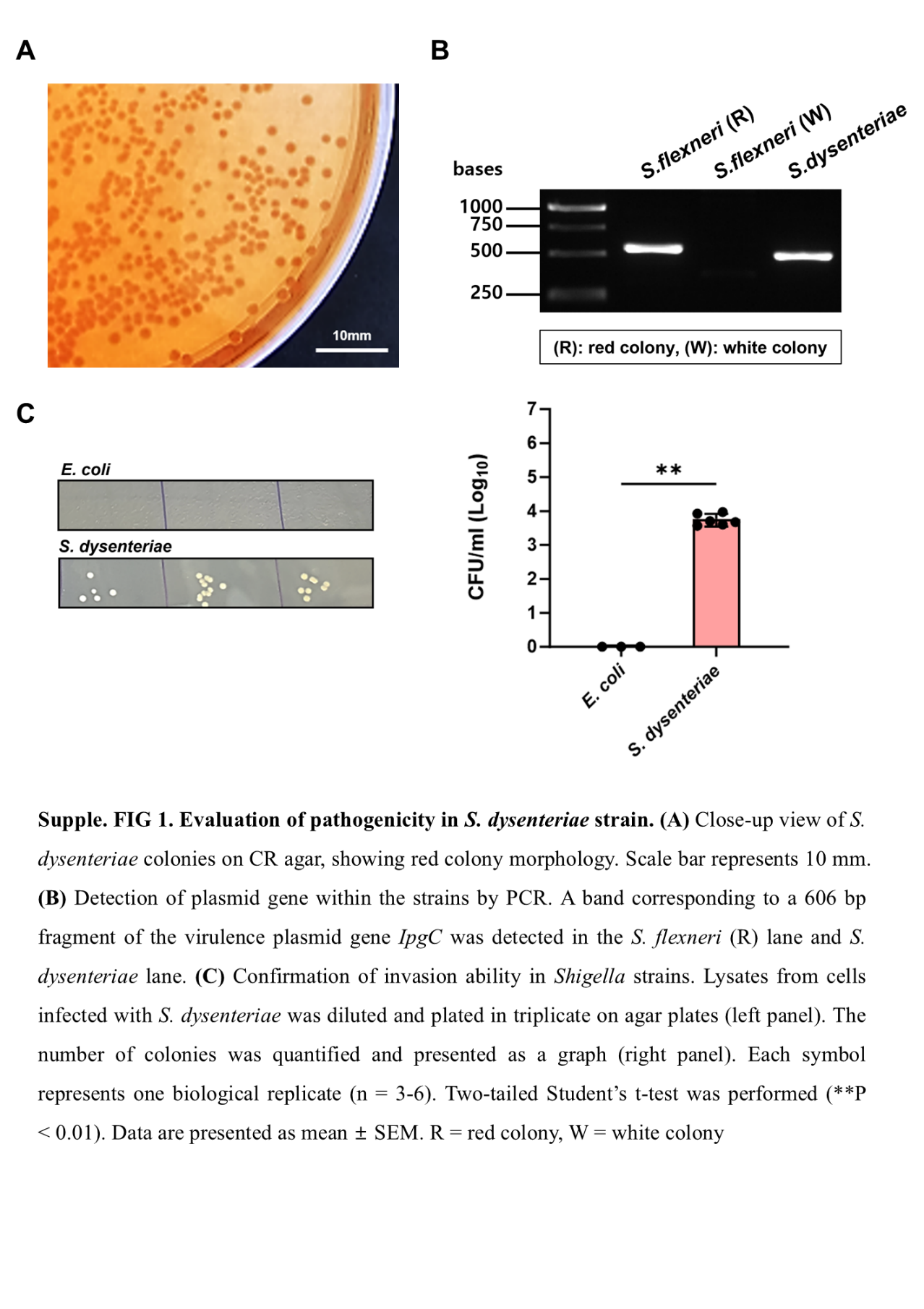


**Supple. Fig. 3.** **Evaluation of pathogenicity in *S. dysenteriae* strain. (A)** Close-up view of *S. dysenteriae* colonies on CR agar, showing red colony morphology. Scale bar represents 10 mm. **(B)** Detection of plasmid gene within the strains by PCR. A band corresponding to a 606 bp fragment of the virulence plasmid gene *IpgC* was detected in the *S. flexneri* (R) lane and *S. dysenteriae* lane. **(C)** Confirmation of invasion ability in *Shigella* strains. Lysates from cells infected with *S. dysenteriae* was diluted and plated in triplicate on agar plates (left panel). The number of colonies was quantified and presented as a graph (right panel). Each symbol represents one biological replicate (n = 3-6). Two-tailed Student’s t-test was performed (**P < 0.01). Data are presented as mean ± SEM. R = red colony, W = white colony


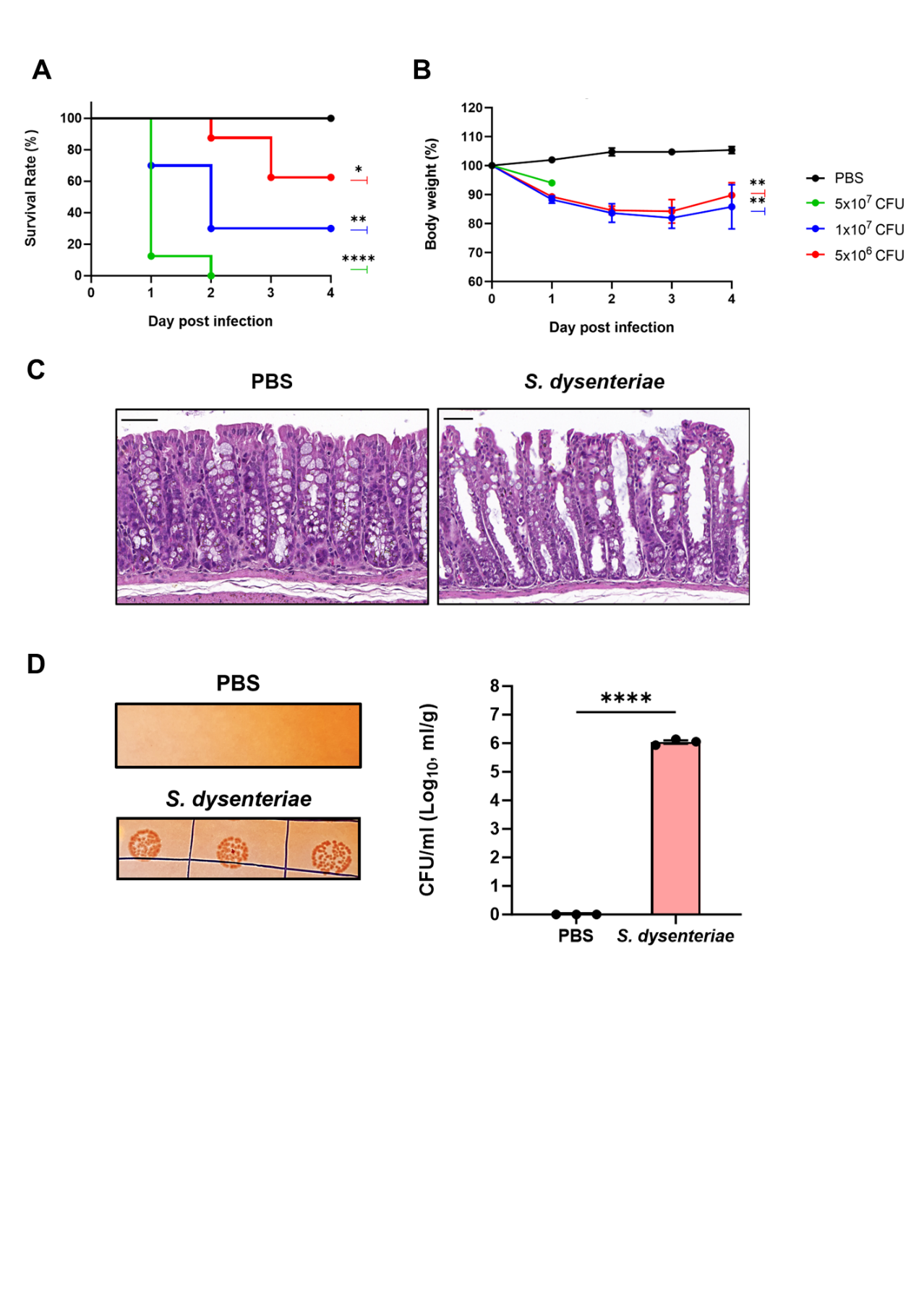


**Supple. Fig. 4. *In vivo* pathogenicity assessment of *S. dysenteriae* strain.** Adult mice were injected via the intraperitoneal (IP) route with three doses (5$\boldsymbol{\times}$10^6^, 1$\boldsymbol{\times}$10^7^, or 5$\boldsymbol{\times}$10^7^ CFU) of *Shigella* strains (8-13 mice per group). Survival rates **(A)** and body weight changes **(B)** were monitored up to 4 days post-infection (dpi). **(C)** Images of H&E-stained colon tissue from infected mice. Scale bar represents 50 μm. **(D)** Confirmation of *Shigella* colonization in the mouse mucosal system. Homogenates of feces from infected mouse were diluted and plated in triplicate on agar plates (left panel). The number of colonies was quantified and presented as a graph (right panel). Each symbol represents two fecal samples from mice (each group; n = 3). Mean ± SEM is shown in (B and D). Statistical analyses were performed using log-rank (Mantel-Cox) test (A), one-way ANOVA with Dunnett’s multiple comparisons test (B), and two-tailed Student’s t-test (D) (ns = not significant, *P < 0.05, **P < 0.01, ***P < 0.001, and ****P < 0.0001 compared to PBS control).
